# Efficacy of intranasal insulin in improving cognition in mild cognitive impairment or dementia: a systematic review and meta-analysis

**DOI:** 10.3389/fnagi.2022.963933

**Published:** 2022-09-12

**Authors:** Cong Long, Xuke Han, Yunjiao Yang, Tongyi Li, Qian Zhou, Qiu Chen

**Affiliations:** ^1^Hospital of Chengdu University of Traditional Chinese Medicine, Chengdu, China; ^2^School of Clinical Medicine, Chengdu University of Traditional Chinese Medicine, Chengdu, China

**Keywords:** dementia, MCI (mild cognitive impairment), cognitive, intranasal insulin, systematic review, meta-analysis

## Abstract

**Background:**

Insulin regulates many aspects of brain function related to mild cognitive impairment (MCI) or dementia, which can be delivered to the brain center via intranasal (IN) devices. Some small, single-site studies indicated that intranasal insulin can enhance memory in patients with MCI or dementia. The pathophysiology of Alzheimer's disease (AD) and diabetes mellitus (DM) overlap, making insulin an attractive therapy for people suffering from MCI or dementia.

**Objective:**

The goal of the study is to evaluate the effectiveness of IN insulin on cognition in patients with MCI or dementia.

**Methods:**

We searched the electronic database for randomized controlled trials (RCTs) that verified the effects of insulin on patients with MCI or dementia.16 studies (899 patients) were identified.

**Results:**

The pooled standard mean difference (SMD) showed no significant difference between IN insulin and placebo groups; however, statistical results suggested a difference between study groups in the effects of ADCS-ADL; AD patients with APOE4 (-) also showed improved performance in verbal memory; other cognitions did not improve significantly.

**Conclusion:**

In view of IN insulin's promising potential, more researches should be conducted at a larger dose after proper selection of insulin types and patients.

**Systematic review registration:**

http://www.crd.york.ac.uk/PROSPERO/, identifier CRD42022353546.

## Introduction

In recent years, a large number of researches has found that Type 2 diabetes mellitus (T2DM) may increase the incidence rate of mild cognitive impairment (MCI) and dementia (Biessels and Despa, 2018). Following 705 participants for 4.6 years, Callisaya et al. indicated that the domains of verbal fluency, verbal memory, and working memory had a greater decline in T2DM patients. Besides, they indicated that T2DM patients had both worse ventricular and brain volume at baseline (Callisaya et al., 2019). T2DM has been confirmed to double the risk of cognitive disorders and dementia, such as in Alzheimer disease (AD) and Vascular dementia (VaD) (Biessels et al., 2006; Kopf and Frölich, 2009). Patients with Parkinson's disease (PD) have nearly six times more chance of developing dementia than their counterparts without PD, according to a study (Aarsland et al., 2017). Additionally, elderly and long-term Parkinson's disease patients are more likely to develop dementia (Hely et al., 2008). Insulin, as an important neurohormone, plays a critical role in brain energy metabolism, cognitive function, axonal migration, and neurogenesis (Chen et al., 2014). Insulin resistance (IR) has been proved to be a pathogenic mechanism of cognitive disorders (Sasaoka et al., 2014). More and more evidence showed that disordered cerebral insulin signaling enhances the development and progression of AD, prompting clinicians to target the loop. Although traditional and contemporary anti-diabetes drugs have shown hope in the fight against insulin resistance (IR), IN insulin seems to be the most effective method to improve brain insulin. IN insulin was more effective than subcutaneous insulin in lowering fasting blood glucose concentrations and in counteracting rises in blood glucose concentrations (Pontiroli et al., 1982). Because of a unique characteristic, insulin can affect the central nervous system (CNS) without passing through the blood-brain barrier. This has been demonstrated to contribute to enhancing the cognitive performance of diabetics, particularly those who have Alzheimer's disease or MCI, as it has been demonstrated that decreasing insulin levels in the brain have a detrimental effect on cognitive function. This helps to reduce the peripheral side effects of insulin in addition to reducing them (Gaddam et al., 2021). Yet, significant open questions remain about the safety, efficacy, and potential of insulin as an adjunct or monotherapy (Chapman et al., 2018). Therefore, this review aims to critically assess the available evidence and future potential of IN insulin as a meaningful treatment for AD and dementia. T2DM has been verified to increase the risk of cognitive disorders and dementia, such as AD and VaD. Besides, there may be a subgroup of dementia related to specific DM-associated metabolic abnormalities (Hanyu, 2019). T2DM is a recognized risk factor for dementia. T2DM and Dementia have some common underlying pathophysiologies, which makes people interested in the reuse of therapeutic drugs for type 2 diabetes, which is beneficial to brain health (Moran et al., 2019). In dementia, there is a progressive deterioration of functional and cognitive abilities, eventually causing a heavy burden on health and social services. The global prevalence of dementia reached 35.6 million in 2010. And the number is expected to double every 20 years (Prince et al., 2013). Insulin increases serum glucose uptake in the peripheral and central nervous system. Abnormal insulin regulation can take place in the early stage of T2DM and dementia, which is a promising therapeutic target to reduce the risk of cognitive disorders (Cha et al., 2016; Arnold et al., 2018). Considering the high concentration of insulin receptors in multiple brain regions associated with dementia, IN insulin inhalation as a treatment for dementia is of great significance, even for people without T2DM (Zhao and Townsend, 2009; Craft, 2012b). In AD patients with or without T2DM, intravenous insulin and glucose normalization can improve memory scores, but peripheral hypoglycemia remains a risk. Contrary to this, IN insulin administration increased brain insulin levels without having a peripheral effect (Craft et al., 1996).

## Materials and methods

### Study registration

This study was registered in PROSPERO (CRD42022353546). This study was constructed according to the guidelines for preferred reporting items for systematic reviews and meta-analyses (PRISMA 2020) (Page et al., 2021).

### Qualification criteria

Qualification criteria for inclusion in the study were as below: Types of studies: Only RCTs were included. Participants: Studies of human participants with MCI or dementia eligible for inclusion, all subjects were free from psychiatric disorders, alcoholism, severe head trauma, hypoxia, neurological disorders other than MCI or dementia, renal or hepatic disease, diabetes, chronic obstructive pulmonary disease, congestive heart failure, or unstable cardiac disease. Interventions: Studies that include receiving any dose of intranasal insulin at any time. Comparator: Any study containing a control group receiving placebo treatment is eligible for inclusion. Outcomes: Studies investigating the efficacy or progression of cognitive disorders or performance (the study reported specific cognitive scores at baseline and endpoints) and dementia (including subtypes of dementia, such as AD or VaD) were eligible for inclusion.

### Information sources

PubMed, Web of Science, Embase, Cochrane Library, Clinical Trials (ClinicalTrials.gov), Chinese Biomedical Literature Database, Chinese National Knowledge Infrastructure (CNKI), Wanfang database were searched for related published studies as of December 20, 2021.

### Search strategy

In the above databases we used the following search query: “(Intranasal insulin OR nasal insulin) AND (Dementia OR neurodegenerative disease OR Cognitive Dysfunction OR cognitive impairment OR neuroprotective OR memory OR cognition).”

### Assessment of methodological quality and data extraction

Critical appraisals were conducted by independent reviewers using the risk of bias (RoB) assessment tool of the Cochrane Collaboration Network assessment checklist (Higgins et al., 2011) for experimental, case-control, cohort, and cross-sectional studies. The consensus was reached through discussion between reviewers. Researches that met >50% of the quality criteria were eligible for selection. Templates used for data extraction, including research method field, study design, data source, inclusion criteria, exposure, country, sample size/event, follow-up, results, result data, and statistical adjustment. When additional data were needed, attempts to contact the corresponding author by e-mail were unsuccessful: in these cases, findings have been summarized narratively.

### Statistical analysis

The combined cognitive performance score change was calculated by the inverse variance method (random effect model) as the standardized mean difference (SMD). Some of the data of these articles (Reger et al., 2006, 2008a,b; Claxton et al., 2013, 2015; Rosenbloom et al., 2014) were obtained from the following article (Lu and Xu, 2019). *X*^2^ test was used for statistical heterogeneity. The Comprehensive Meta-analysis software (version 3.3) and Review Manager (Revman, version 5.3) were used for data analysis.

## Results

### Search results

**Figure 1:**
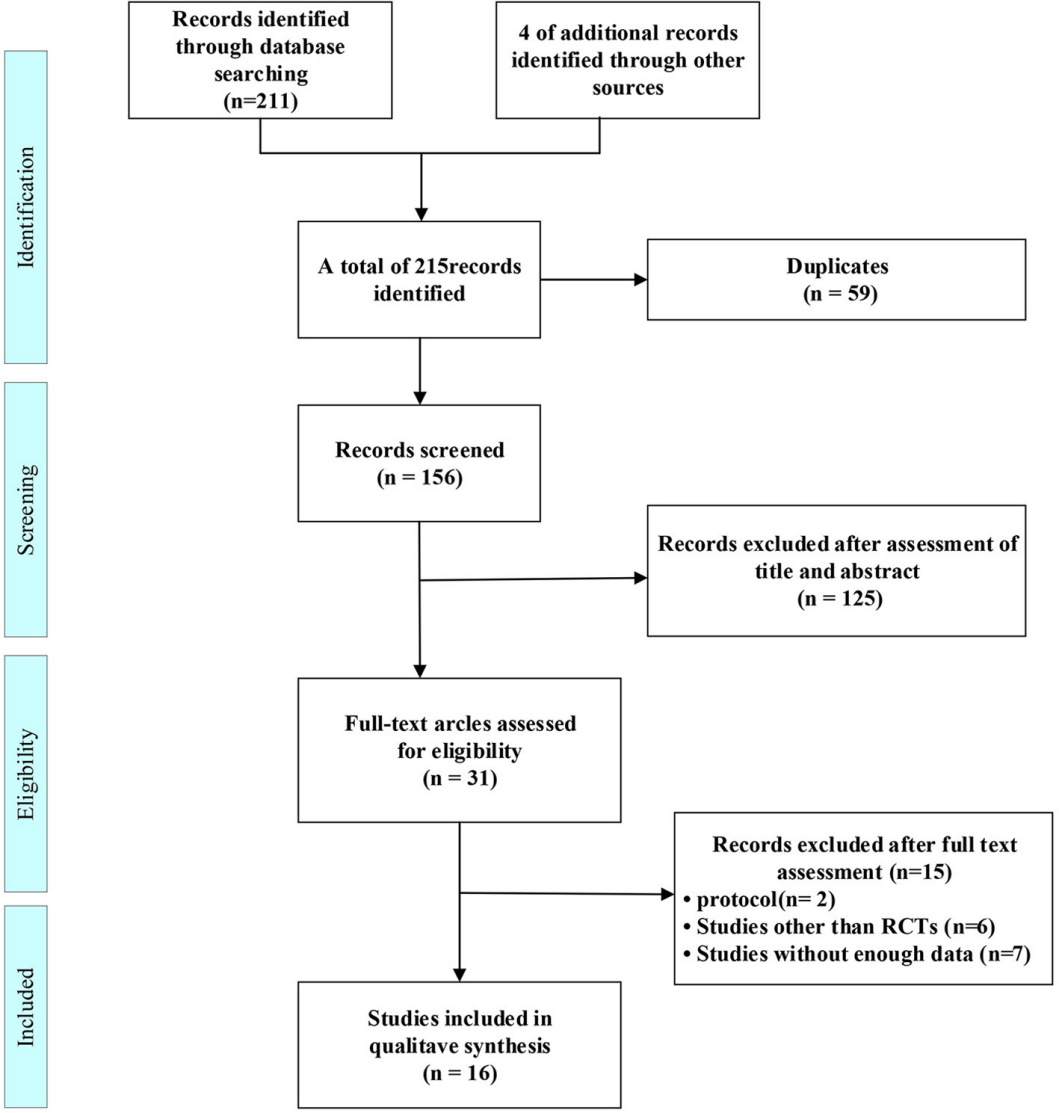
The flowchart of the study selection process.

215 citations were found in the original search, which were reduced to 156 after duplicates were eliminated. 125 studies were identified after titles and abstracts were reviewed. The full-text search and 31 studies were qualified. a further 15 studies were excluded for protocol (Nct, 2021; Nitchingham et al., 2021), studies other than RCTs (Bayés et al., 2006; Reger and Craft, 2006; Xiaojiu and Xuan, 2015; Yanfang, 2016; Deng Yun, 2020; Tashima, 2020), or without enough data (Nct, 2007, 2012, 2019; Anonymous, 2008; Galindo-Mendez et al., 2020; Gwizdala et al., 2021; Roque et al., 2021) leaving 16 included studies (Figure 1).

### Studies' and patients' characteristics

Three crossover studies (Rosenbloom, 2011; Rosenbloom et al., 2014; Cha et al., 2017) out of the 16 papers we analyzed were included in our meta-analysis, Two studies used a parallel design (Craft, 2012a; Craft et al., 2020). The follow-up period is from 1 day to 72 weeks. The sample size ranged from 9 to 240. One study was HIV Dementia (Rubin, 2017), one was diagnosed with MDD(major depressive disorder) (Cha et al., 2017), and two studies enrolled patients with PDD (Parkinson's disease Dementia) (Novak et al., 2019; Yufeng, 2020), the others were about AD or MCI (Reger et al., 2006, 2008a,b; Rosenbloom, 2011; Craft, 2012a,b; Claxton et al., 2013, 2015; Rosenbloom et al., 2014, 2021; Craft et al., 2017; Yufeng, 2020). These studies were conducted in the following regions: Canada (*n* = 1), China (*n* = 1), America (*n* = 14). The publication year of the included articles ranged from 2006 to 2021. The mean age of patients ranged from 18 to 90 years. The baseline characteristics of the eligible studies are shown in Table 1.

**Table 1:** Summary of general characteristics of the included studies.

**References**	**Country**	**Study design**	**Number and patients, characteristics**	**Treatment duration (days)**	**Cognitive test used**	**Adverse event**
Reger et al. (2006)	USA	Randomized no blinding is indicated	• 35 normal adults (27 ε4- and 8 ε4+) • 13 AD patients (6 ε4- and 7 ε4+) • 13 MCI patients (8 ε4- and 5 ε4+)	15 min	Story recall Buschke Selective Reminding. • Test self-ordered pointing task • Stroop color-word test visual search task	Minor nosebleed (1), nose soreness (1)
Reger et al. (2008b)	USA	Randomized placebo- controlled, pilot clinical trial (all participants were blinded)	• 25 subjects • AD or aMCI	21 days	• Memory savings scores • Stroop voice onset times errors for concordant and discordant trials • DSRS scores	Headache (1), nasal dripping (2), weakness (1), sneezing (1), blood glucose between 60 and 70 mg/dL (1)
Reger et al. (2008a)	USA	Randomized the study was not blinded	• 33 patients (11 ε4- and 22 ε4+) with either: probable AD (13) or MCI or multiple domain MCI with amnestic features (20) • 59 normal adults (48 ε4–and 11 ε4+)	15 min	• Story Recall and Hopkins Verbal Learning Test • Self-Ordered Pointing Task	Not mentioned
Rosenbloom (2011)	USA	Randomized, double-blind, placebo-controlled, cross-over designed	12 adults with AD	112 days	• Cognitive Performance • Trails B - Seconds • Trails B - Errors • Olfactory Function	No treatment related severe adverse events occurred
Craft (2012a)	USA	Randomized, double-blind, placebo-controlled trial	• 104 adults • AD = 40 • aMCI = 64	120 days	• Delayed story recall score • DSRS score • ADAS-cog • ADCS-ADL	No treatment related severe adverse events occurred
Craft (2012b)	USA	RCT parralle	36 adults with AD or MCI	120 days	• Executive Function Composite (Sum of Z Scores from Dot Counting Test and Benton Visual Retention Test Form F&G) • ADAS-Cog	No treatment related severe adverse events occurred
Claxton et al. (2013)	USA	Randomized clinical trial (all participants were blinded)	• 104 patients, with MCI (64) or AD (40) • ε4-: 32 men and 25 women • ε4+: 27 men and 20 women	120 days	• DSRS • ADAS-Cog • ADCS-ADL	No treatment related severe adverse events occurred
Rosenbloom et al. (2014)	USA	Phase II, double-blinded, randomized, crossover study	9 mild-to-moderate AD patients, ε4+	14 ± 3 days	• RBANS • WAIS-IV • BNT • Trail-Making Test	No treatment related severe adverse events occurred
Claxton et al. (2015)	USA	Pilot, randomized controlled trial (blinded)	60 older adults: 39 with and 21 with probable AD	21 days	Verbal memory composite score (the sum of z scores of immediate and delayed story recall and immediate delayed word list recall) verbal working memory (Dot Counting N-back)	No treatment related severe adverse events occurred
Cha et al. (2017)	USA	Randomized, double-blind, placebo-controlled trial	36 adults with AD or MCI	120 days	• Composite memory score (sum of Z scores for delayed list and story recall) • ADAS-Cog • DSRS, MRI volume changes in AD- related regions of interest, and cerebrospinal fluid AD markers	No treatment related severe adverse events occurred
Rubin (2017)	USA	Randomized double-blind placebo-controlled clinical trial	21 adults with HIV Dementia	168 days	• GDS • NPZ-8 Score	Cardiac event (1),Kidney obstruction (1), Hospitalization for syncopehypotension (1)
Craft et al. (2017)	Canada	Randomized, double-blind, placebo-controlled, crossover trial	35 adults with Cognitive dysfunction in MDD (major depressive disorder)	90 days	• AGN (Correct Response) • ERT (Correct Response) • ERT (Response Time in ms)	Not mentioned
Novak et al. (2019)	USA	Proof of concept randomized, double-blinded, placebo-controlled trial	14 adults with Cognitive impairment in PD	28 days	• MoCA • HY classification • BDI • FAS total • UPDRS	No treatment related severe adverse events occurred
Yufeng (2020)	China	RCT	15 adults with PD-CI	28 days	• MMSE • MoCA • UPDRS	No treatment related severe adverse events occurred
Craft et al. (2020)	USA	Randomized parallel assignment	240 adults with AD or aMCI	504 days	• ADAS-Cog • MMSE • ADCS-ADL-MCI • CDR-SB • NPI score	No treatment related severe adverse events occurred
Rosenbloom et al. (2021)	USA	Single-center, randomized, double-blind, placebo-controlled study	• 35 adults with AD or aMCI • 50–90 years	224 days	• ADAS-Cog13 • CDR-SOB • FAQ scores	No treatment related severe adverse events occurred

DSRS, Dementia Severity Rating Scale; ADAS-cog, Alzheimer Disease's Assessment Scale–cognitive subscale; ADCS-ADL, Alzheimer's Disease Cooperative Study–activities of daily living; RBANS, Repeatable Battery for the Assessment of Neuropsychological Status; WAIS-IV, Wechsler Adult Intelligence Scale—Fourth Edition; BNT, Boston Naming Test, GDS, Global Deficit Score; NPZ-8 Score, 8 neurocognitive performance individual Z-scores; MoCA, Montreal Cognitive Assessment; UPDRS, Unified Parkinson's Disease Rating Scale; MMSE, Mini-mental State Examination; HY classification, Hoehn and Yahr scale; BDI, Beck Depression Inventory; FAS, phonemic fluency and verbal memory; NPI, Neuropsychiatric Inventory; FAQ, Functional Activities Questionnaire.

### Risk of bias

**Figure 2:**
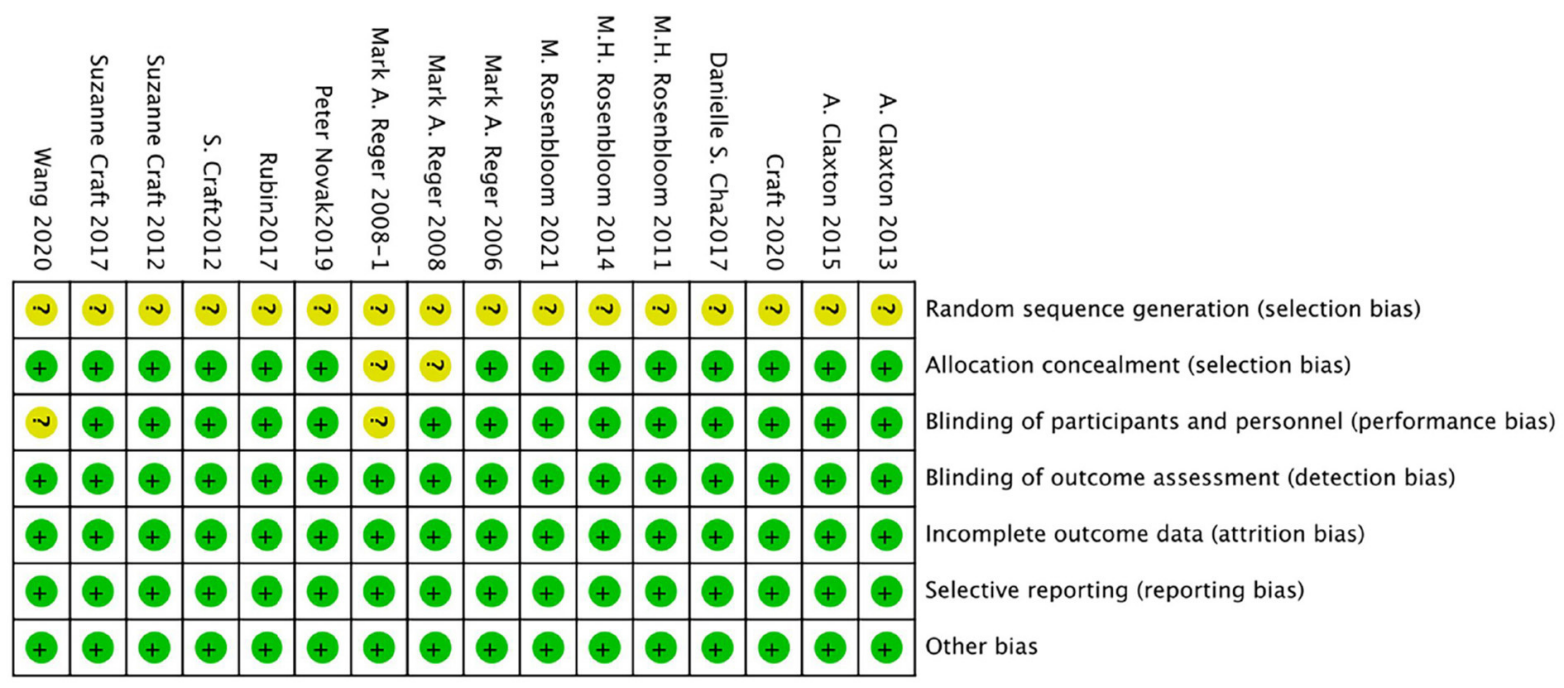
Risk of bias summary for each risk of bias item for each included study.

**Figure 3:**
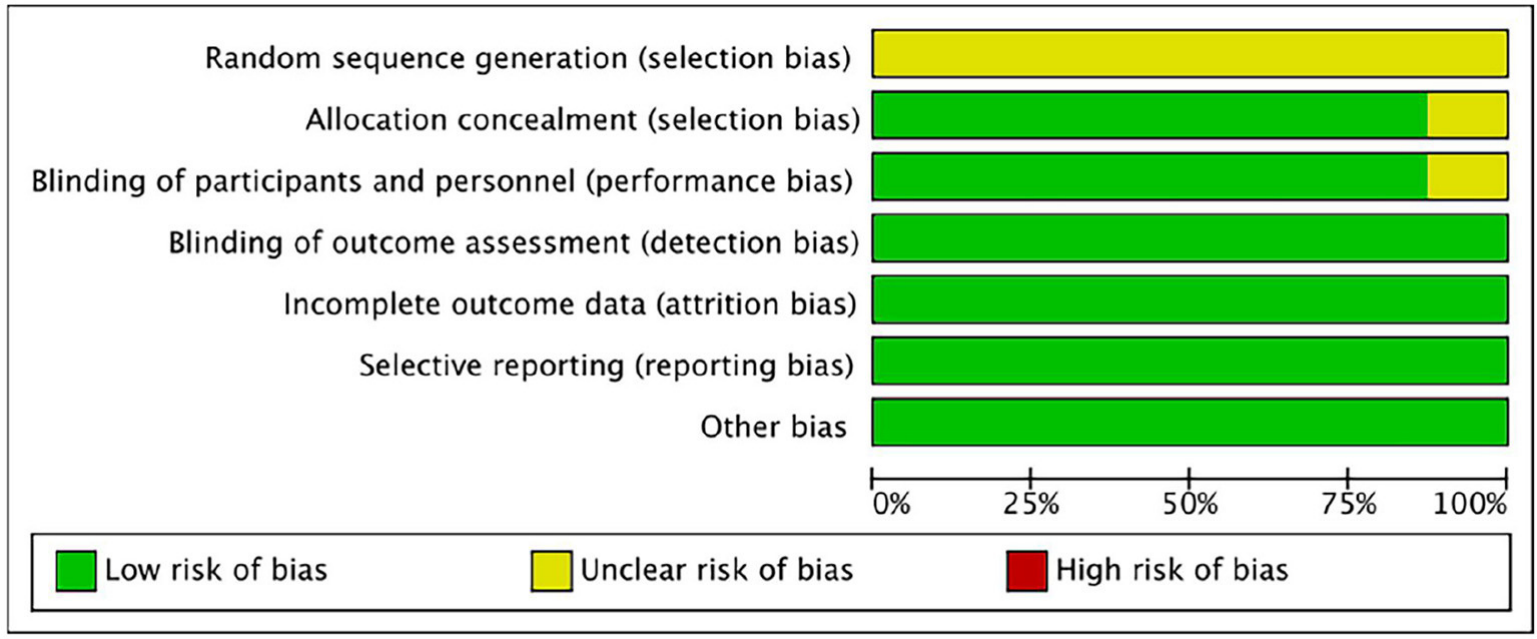
Risk of bias graph authors' judgments about each risk of bias item, presented as a percentage across all induded studies.

The bias risk of the randomized controlled trial was independently assessed by two reviewers (Yunjiao Yang and Tongyi Li) using the bias risk assessment tool of the Cochrane Collaboration Network (RoB). All of the researches were deemed to be low risk in the evaluation. For all studies, the risk of “random sequence generation” is unclear, and the RoB is unclear; The RoBs of the four studies were not clear because the risks of “blinding of participants” and/or “allocation concealment” were not clear (Reger et al., 2006, 2008a,b; Novak et al., 2019). See Figures 2, 3 for RoB evaluation details. A cross-over study of Rosenbloom et al. was identified as low risk due to the design of the study (AD is a disease with a persistent course), randomization of the treatment sequence resulted in no subsequent treatment effect.

### Publication bias

**Figure 4:**
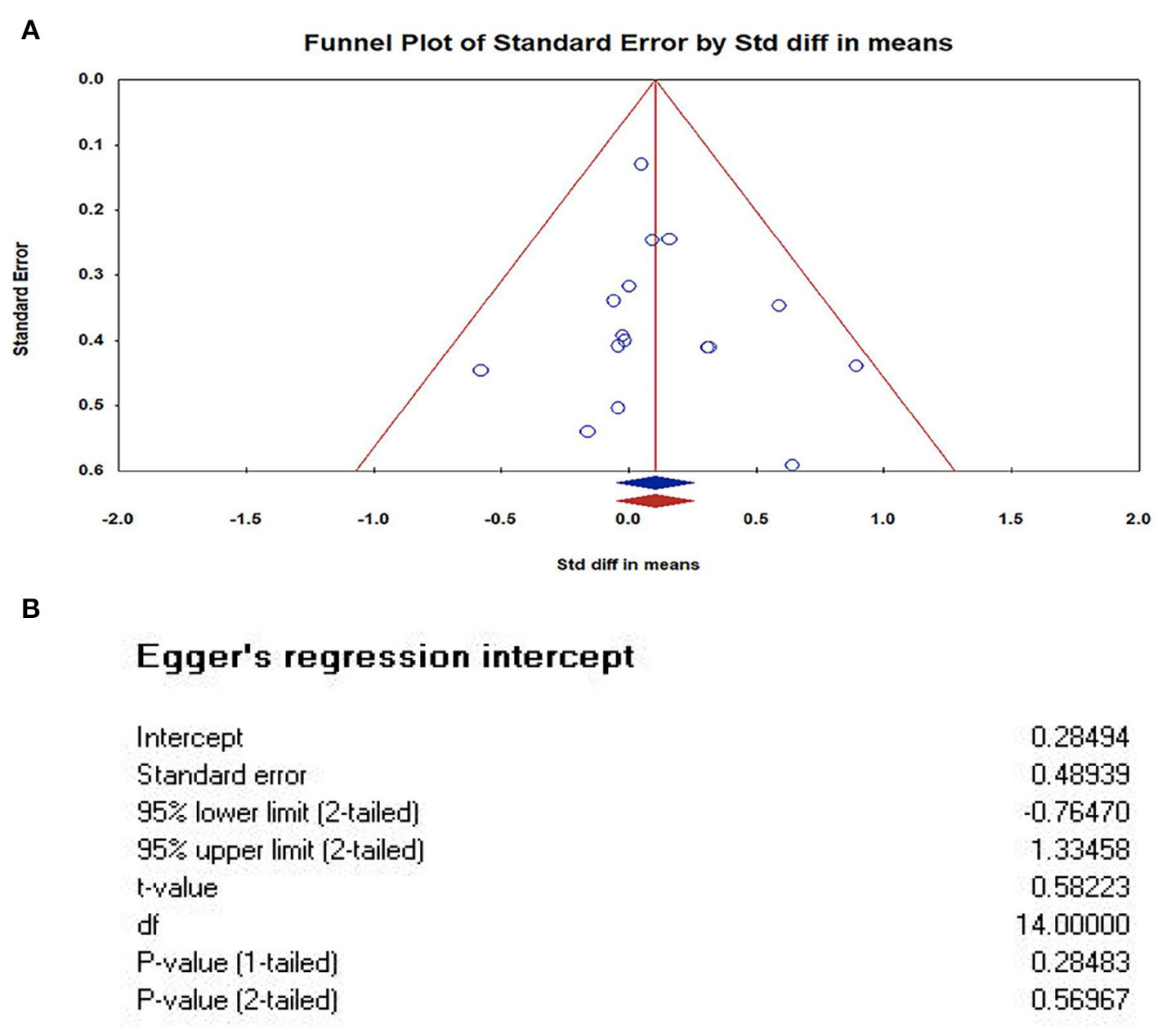
Publication bias of studies. **(A)** Funnel Plot of Standard Error by Std diff in means (Plot observed and imputed); **(B)** The results of Egger's tests. In this case the intercept (BO) is 0.28494, 95% confidence interval (−0.76470, 1.33458), with *t* = 0.58223, *df* = 14. The 1-tailed *p*-value (recommended) is 0.28483, and the 2-tailed *p*-value is 0.56967. The results of Egger's tests indicates that there is no publication bias.

We used The Comprehensive Meta-analysis software (version 3.3) to analyze and make a funnel plot, as shown in Figure 4. In order to further test publication bias, we further used Egger' test. The results of Egger's tests indicates that there is no publication bias. In this case the intercept (B0) is 0.28494, 95% confidence interval (−0.76470, 1.33458), with *t* = 0.58223, *df* = 14. The 1-tailed p-value (recommended) is 0.28483, and the 2-tailed p-value is 0.56967.

### Cognitive performance

#### Combined cognitive performance outcome

**Figure 5:**
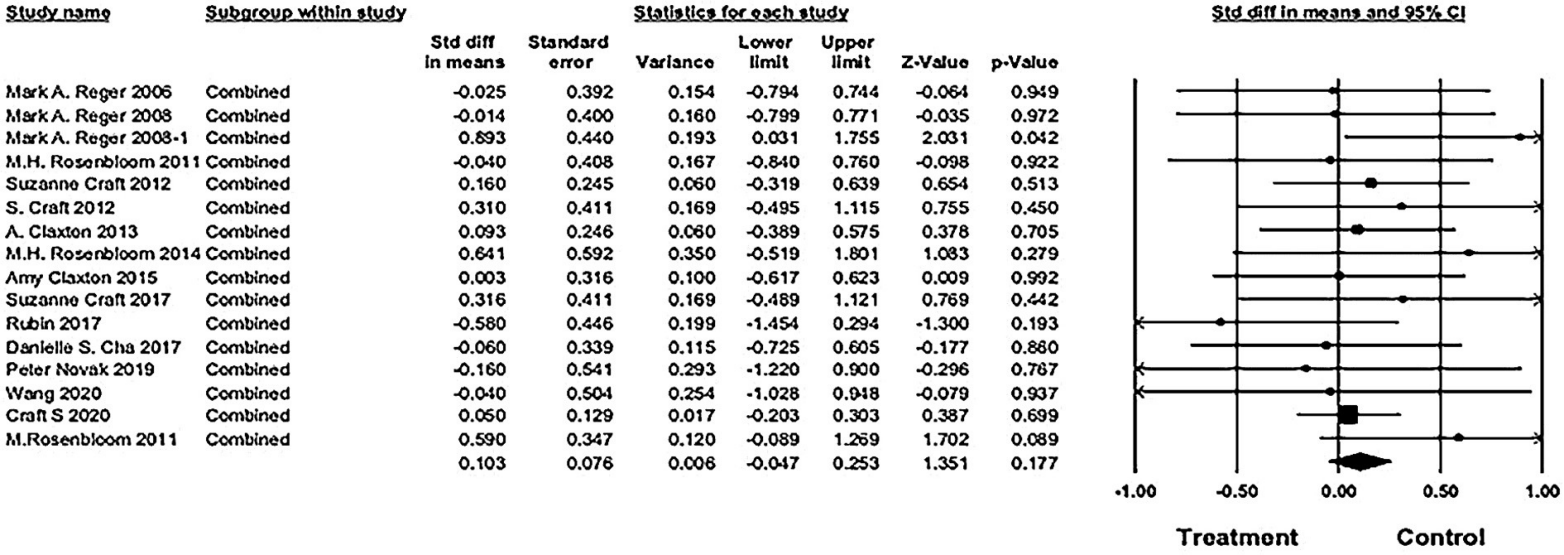
Forest plot of pooled standard mean difference (SMD) for combined cognitive score. The pooled SMD was 0.103 [16 studies; 95% confidence interval (CI), −0.05 to 0.25; *P* = 0.18), showing no significant difference between IN insulin and placebo groups (weights and heterogeneity test are from random-effects model).

The pooled SMD was 0.103 [16 studies; 95% confidence interval (CI), −0.05 to 0.25; *P* = 0.18], showing no significant difference between IN insulin and placebo groups (see Figure 5).

#### Effects of ADAS-cog

**Figure 6:**
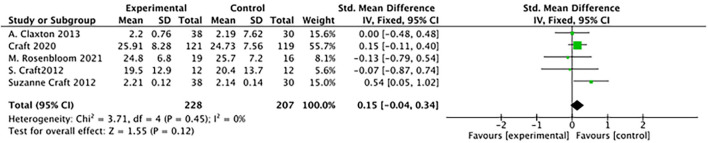
Forest plot for the Effects of ADAS-cog (Alzheimer's Disease's Assessment Scale–cognitive subscale). There was no difference between study groups: SMD = 0.15, 95% CI = −0.04 to 0.34; *P* = 0.12).

There was no difference between study groups: (5 studies: SMD = 0.15, 95% CI= −0.04 to 0.34; *P* = 0.12; see Figure 6).

#### Effects of ADCS-ADL, MMSE, MoCA, UPDRS

**Figure 7:**
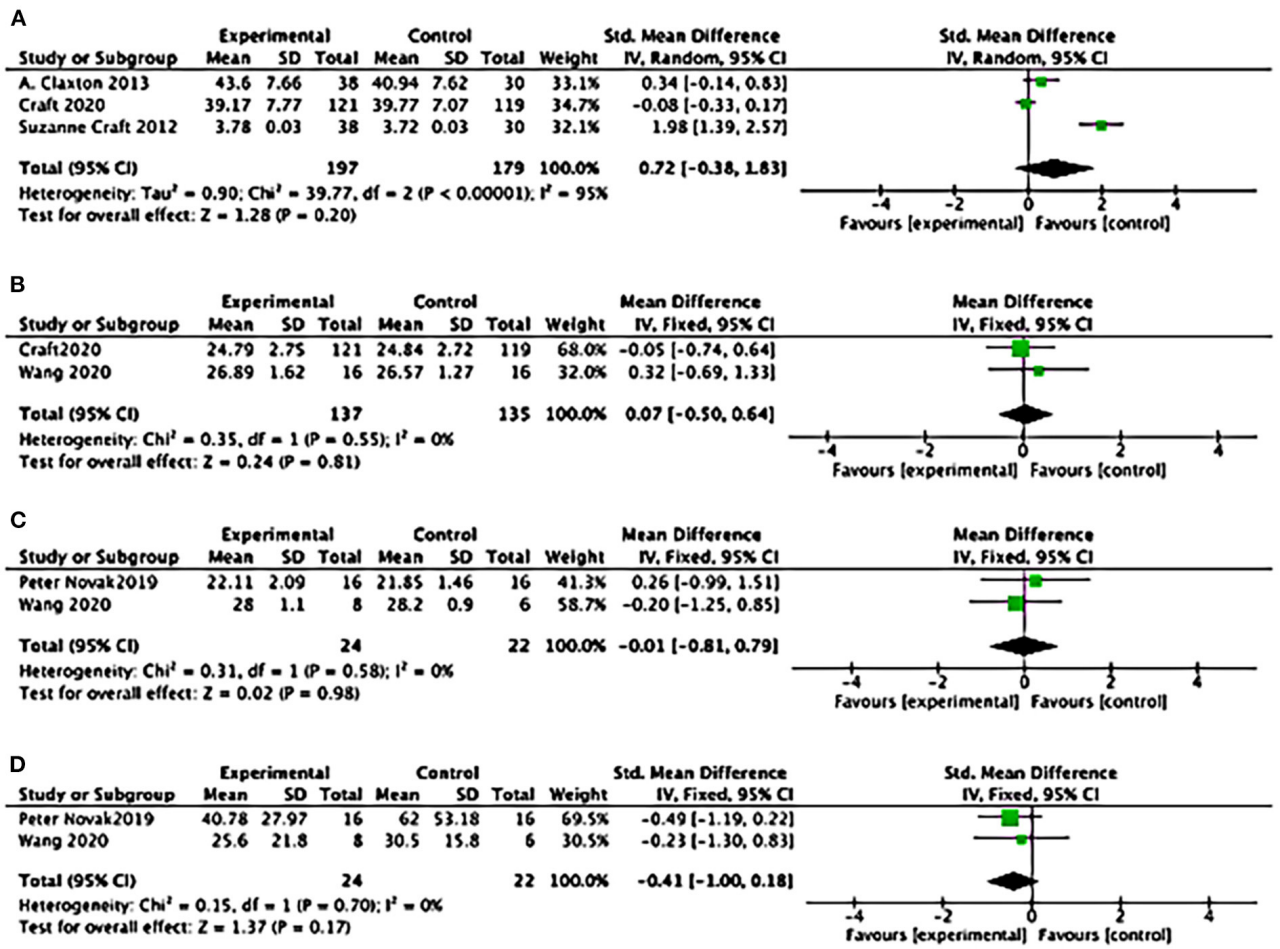
Forest plot for the effects of cognitive performance. **(A)** Forest plot for the effects of the Alzheimer's Disease Cooperative Study–activities of daily living Statistical (ADCS-ADL): results suggested a difference between study groups: (3 studies): SMD = 0.26, 95% CI = 0.05 to 0.47; *P* = 0.01); **(B)** Forest plot for the effects of Mini-mental Atate Examination (MMSE): SMD favoured Insulin but not statistically different of the effects (2 studies): SMD = 0.07, 95% CI = −0.50 to 0.64; *P* = 0.81; **(C)** Forest plot for the effects of Montreal Cognitive Assessment (MoCA): No differences between study groups of the effects (2 studies): SMD = −0.01, 95% CI = −0.81 to 0.79; *P* = 0.98; **(D)** Forest plot for the effects of Unified Parkinson's Disease Rating Scale (UPDRS): There was no difference between study groups (2 studies): SMD = −0.41, 95% CI = −1.00 to 0.18; *P* = 0.17).

Statistical results suggested a difference of ADCS-ADL between study groups: (3 studies): SMD = 0.26, 95% CI = 0.05–0.47; *P* = 0.01) (see Figure 7A); SMD favored insulin but not statistically different of the effects of MMSE (2 studies): SMD = 0.07, 95% CI = −0.50 to 0.64; *P* = 0.81; see Figure 7B); No differences between study groups of the effects of MoCA (2 studies): SMD= −0.01, 95% CI = −0.81 to 0.79; *P* = 0.98; see Figure 7C); There was no difference between study groups of the effects of UPDRS (2 studies: SMD = −0.41, 95% CI = −1.00 to 0.18; *P* = 0.17; see Figure 7D).

#### Effects modulation by APOE genotype

In the study (Reger et al., 2006) insulin treatment facilitated recall on two measures of verbal memory in memory-impaired ε4– adults. Findings in this study (Reger et al., 2008a) suggests that IN insulin administration dose-dependently modulates verbal memory, the acute clinical benefits of treatment were greatest at 20 IU.

#### Effects of Aβ42 and Aβ40

In two studies, IN insulin was found to affect plasma amyloid levels Aβ42 and Aβ40 (Reger et al., 2008a,b). Another two studies also cited the effects on CerebroSpinal Fluid(CSF) Aβ42 and Aβ40 levels (Craft, 2012b; Craft et al., 2017). Rather contradictory results emerged.

### Metabolic data

In four studies, IN insulin therapy was shown to affect plasma glucose and insulin levels (Reger et al., 2006, 2008a,b; Rosenbloom et al., 2014). Blood glucose and insulin levels in three of these studies did not change significantly after treatment (Reger et al., 2006, 2008a; Rosenbloom et al., 2014). After 21 days of treatment, fasting glucose or insulin levels did not change in Rosenbloom et al.'s study. In spite of this, postprandial plasma insulin levels decreased in the treatment group when compared to the placebo group [*F*_(1, 20)_ = 4.43, *P* = 0.0481)] (Rosenbloom et al., 2014).

## Discussion

### Main findings and conclusions

Based on our current study, there was no significant effects of intranasal insulin on the improvement of cognitive function in patients with MCI/dementia. However, Even though the results were not statistically significant, this study illustrates the potential of IN insulin efficacy. According to Mark A Reger's work, insulin dose-response curves vary by APOE genotype and IN insulin treatment may offer therapeutic benefits without the danger of peripheral hypoglycemia, the effect of APOE genotype on cognitive and metabolic responses to insulin may reflect a specific pattern of abnormal insulin metabolism among ε4– subjects, differential cognitive responses to insulin treatment by APOE genotype may result from differences in insulin sensitivity (Reger et al., 2006, 2008a). There is growing evidence that persons with AD who do not carry ApoE-ε4 may experience IR more frequently. Higher doses of insulin are needed to elicit biological reactions that are typically seen at lower insulin doses in healthy persons with IR, which is a condition in which muscle, fat, and hepatic cell responses to insulin are impaired. But evidence is still lacking to explain the mechanism by which the ApoE4 genotype attenuates the cognitive response to IN insulin. Statistical results also revealed a difference between study groups in the effects of ADCS-ADL. We also find some subtle but meaningful patterns of improvement in cognitive function with intranasal insulin. For example, the type of intranasal insulin, the treatment course and the device used will affect the treatment, and these studies suggest potential mechanisms of intranasal insulin in MCI or dementia treatment: change brain energy metabolism, modulates plasma Aβ and cortisol levels, stabilize or improve cerebral glucose metabolism, change insulin signal in the CNS, modifying AD-related pathophysiologic processes, protect hippocampal neurons against oxidative stress and apoptotic cell death, increase resting-state functional connectivity between hippocampal and Default Mode Network(DMN) regions (see Table 2). However, due to the lack of sample size, the amount of data for specific analysis is not enough. In the future, we will continue to pay attention in this field to see if we can dig out some specific patterns of intranasal insulin treatment.

**Table 2:** Characteristics of included studies.

**References**	**Treatment regimens (acute or chronic)**	**Patient diagnosis**	**Therapeutic device**	**Type and dose of insulin**	**Possible impact mechanism**
Reger et al. (2006)	Acute	MCI or AD	A needle-less syringe	• Novolin R • 20 or 40 IU	Change brain energy metabolism
Reger et al. (2008b)	Acute	MCI or AD	An electronic atomizer	• Novolin R • 20IU Bid	• Modulates plasma • Aβ and cortisol levels
Reger et al. (2008a)	Acute	MCI or AD	A needle-less syringe	• Novolin R • 10, 20, 40, 60 IU	Change brain energy metabolism
Rosenbloom (2011)	Chronic	AD	MAD 300 device	• Glulisine • 20IU	Not mentioned
Craft (2012a)	Chronic	MCI or AD	• A nasal drug delivery device • Kurve Technology	• Insulin • 20IU/40IU	Stabilize or improve cerebral glucose metabolism
Craft (2012b)	Chronic	MCI or AD	Not mentioned	• Detemir/Novolin R • 40IU	Change insulin signaling in the CNS
Claxton et al. (2013)	Chronic	MCI or AD	A nasal delivery device	• Insulin • 20IU or 40IU	Change brain energy metabolism
Rosenbloom et al. (2014)	Acute	AD	LMA mucosal atomization device (MAD)	• Rapid-acting IN insulin glulisine • 20 IU	No significant effect on cognitive outcome
Claxton et al. (2015)	Chronic	MCI or AD	• ViaNase nasal drug delivery device • Kurve Technology	• Detemir • 20 or 40 IU	No significant effect on cognitive outcome
Cha et al. (2017)	Chronic	MCI or AD	A nasal delivery device	• Detemir/regular • 40IU	Modifying AD-related pathophysiologic processes
Rubin (2017)	Chronic	HIV Dementia	Insulin modifying therapy (IMT)	• Novolin R • 40IU	Protect hippocampal neurons against oxidative stress and apoptotic cell death
Craft et al. (2017)	Chronic	Major depressive disorder (MDD)	Administered via puffs	• Humulin R • 40IU 4 times	No significant effect on cognitive outcome
Novak et al. (2019)	Chronic	PD	• Nase device • Kurve technology	• Novolin R • 40IU	Increase resting-state functional connectivity between hippocampal and DMN regions
Yufeng (2020)	Chronic	PD	Not mentioned	• Insulin aspart • 20IU	Improve cognitive function of PD patients by regulating DMN
Craft et al. (2020)	Chronic	MCI or AD	• Device1 (ViaNase) • Device 2 (I109 Precision Olfactory Delivery)	• Humulin-RU-100 • 40IU	No cognitive or functional benefits were observed
Rosenbloom et al. (2021)	Chronic	MCI or AD	Impel NeuroPharma I109 Precision Olfactory Delivery Device	• Glulisine • 40IU	No enhancing effects of intranasal glulisine on cognition, function, or mood

Abbreviations: DMN regions:Default Mode Network(DMN) regions.

In light of those promising results, further studies with larger sample sizes and longer duration are needed to verify these findings. The effects of IN insulin on MCI/dementia needs to highlight in future studies. With the rapid increase in the aging population, effective treatments for MCI/dementia are in short supply. Since MCI and dementia involve the imbalance of neuropeptide signals in the brain, neuropeptide in management may be a promising therapeutic target, directly aiming at the brain and restoring signals, and ultimately, cognition. In insulin is expected to be a successful treatment for MCI/dementia.

## Limitations

In insulin may be a new therapy for patients with MCI or dementia, but for now, it has only been tested in a few clinical trials, particularly for the treatment of dementia. In our study, there only included 899 patients in all. No safe conclusions can be drawn from such a small sample size. Further, there are restrictions on the properties that can be attributed to the results given that most included studies were conducted in one country (the United States). The heterogeneity of the included studies is another limitation of this research. The first factor that was heterogeneous was the patients' characteristics, particularly their type of dementia, ApoE4 status, and cognitive tests (see Table 1). Moreover, different researches have used different types and doses of insulin (see Table 1). As a final point, the treatment time was uneven (the treatment days ranged between 15 min and 504 days). Therefore, it is not possible to conduct quantitative analysis (meta-analysis) of the included studies.

## Data availability statement

The original contributions presented in the study are included in the article/supplementary material, further inquiries can be directed to the corresponding author/s.

## Author contributions

CL and QC proposed the idea and designed the whole research, literature research, study selection,. and data extraction were conducted by XH, TL, YY, and QZ. The article was written and revised by CL. All authors approved the final manuscript before submission.

## Funding

This study was supported by the Sichuan Science and Technology Program (2019YFS0085). The funder was not involved in the study design, collection, analysis, interpretation of data, the writing of this article or the decision to submit it for publication.

## Conflict of interest

The authors declare that the research was conducted in the absence of any commercial or financial relationships that could be construed as a potential conflict of interest.

## Publisher's note

All claims expressed in this article are solely those of the authors and do not necessarily represent those of their affiliated organizations, or those of the publisher, the editors and the reviewers. Any product that may be evaluated in this article, or claim that may be made by its manufacturer, is not guaranteed or endorsed by the publisher.
